# Local Regrowth After Neoadjuvant Chemoradiotherapy and Active Surveillance for Esophageal Cancer: An Analysis of Surgical Specimens From the SANO Trial

**DOI:** 10.1097/SLA.0000000000007179

**Published:** 2026-08-21

**Authors:** Sanjiv S. G. Gangaram Panday, Lindsey Oudijk, Sjoerd M. Lagarde, Bianca Mostert, A. Stijn L. P. Crobach, Ilse Diederen, Heleen Doornewaard, Karen E. Hamoen, Liudmila L. Kodach, Ineke van Lijnschoten, Judith Nieken, Chella S. van der Post, Robert Riedl, Arjan van Tilburg, Manon C. W. Spaander, J. Jan B. van Lanschot, Michail Doukas, Bas P. L. Wijnhoven

**Affiliations:** 1 Department of Surgery, Erasmus MC Cancer Institute, University Medical Center, Rotterdam, the Netherlands; 2 Department of Pathology, Erasmus MC, University Medical Center, Rotterdam, the Netherlands; 3 Department of Medical Oncology, Erasmus MC Cancer Institute, University Medical Center, Rotterdam, the Netherlands; 4 Department of Pathology, Leiden University Medical Center, Leiden, the Netherlands; 5 Department of Pathology, LabPON, Almelo, the Netherlands; 6 Department of Pathology, Gelre Hospital, Apeldoorn, the Netherlands; 7 Department of Pathology, Maasstad Hospital, Rotterdam, the Netherlands; 8 Department of Pathology, The Netherlands Cancer Institute, Amsterdam, the Netherlands; 9 Department of Pathology, PAMM, Eindhoven, the Netherlands; 10 Department of Pathology, Pathology Friesland, Leeuwarden, the Netherlands; 11 Department of Pathology, Radboud University Medical Center, Nijmegen, the Netherlands; 12 Department of Pathology, Zuyderland Medical Center, Heerlen, the Netherlands; 13 Department of Pathology, Reinier de Graaf Gasthuis, Delft, the Netherlands; 14 Department of Gastroenterology and Hepatology, Erasmus MC University Medical Center, Rotterdam, the Netherlands

**Keywords:** esophageal cancer, neoadjuvant chemoradiotherapy, anatomic layer, local regrowth

## Abstract

**Objective:**

To assess pattern of regrowth after neoadjuvant chemoradiotherapy (nCRT) for esophageal cancer. This may inform optimization of response evaluations and explore the possibility for endoscopic treatment of cancer regrowth.

**Summary Background Data:**

Active surveillance is a treatment approach for patients with locally advanced esophageal cancer but the timely detection of regrowth remains challenging.

**Methods:**

A retrospective cohort study was conducted in patients with complete clinical response 3 months after nCRT who underwent active surveillance. Patients with cancer regrowth that underwent surgery were included. Resection specimens were reviewed by 2 pathologists. The primary outcome was the anatomic layer(s) of regrowth within the esophageal wall.

**Results:**

In total, 75 resection specimens were available 6–36 months after nCRT. Cancer cells were seen in the mucosal layer in 68 of 75 (91%) patients. In 4/75 (5%) patients the tumor was confined to the mucosa. In 13/75 patients (17%), tumor was seen in the mucosa and submucosa but 4 patients also had lymph node involvement. Overall, tumor was present in the submucosa in 88%, proper muscle layer in 71%, and surrounding stroma in 61% of specimens. The majority of regrowth occurred in all layers and this pattern did not change over time.

**Conclusions:**

Detected regrowth after nCRT predominantly involves the mucosa and submucosa in the majority of patients and its temporal regrowth pattern support endoscopy as a diagnostic tool. Given the risk of undetected nodal disease and the current limitations of staging after nCRT, surgery remains the recommended treatment for regrowth.

Active surveillance after neoadjuvant chemoradiotherapy (nCRT) is noninferior to standard surgery for 2-year overall survival as shown in the Surgery As Needed for Oesophageal cancer (SANO) trial. However, long-term follow-up is needed to further evaluate its safety.^1^ Within this context, diagnostic tests used for response assessment during active surveillance are crucial to detect cancer regrowth before metastases occur or the tumor becomes locally irresectable.

The pre-SANO trial assessed the accuracy of diagnostic modalities used for the detection of residual disease. A sensitivity of 77% for the combination of endoscopy and endoscopic ultrasound (EUS) to detect a complete pathologic response at 3 months after nCRT was reported.^2^ Endoscopy with more rigorous bite-on-bite biopsies showed a 20% higher detection rate due to an increased number of biopsies rather than deeper tissue sampling.^3^ In the SANO trial, 46% of patients who initially had complete clinical response (CCR) at 3 months after nCRT developed locoregional regrowth within 2 years.^1^ This shows that current diagnostic tests used in active surveillance are still suboptimal and need improvement.

Insight in the pattern of regrowth in the esophageal wall in patients undergoing active surveillance may help to develop more accurate endoscopic or imaging strategies. If local regrowth is restricted to the mucosa or superficial submucosal layer, this may be treated with endoscopic resection in well-selected patients increasing the number of patients that retain their esophagus.^4–9^

This study aims to assess the location of cancer regrowth within the esophageal wall in patients that underwent active surveillance and to assess if endoscopic resection for cancer regrowth may be a feasible treatment option.

## METHODS

### Study Design and Patients

A retrospective analysis was conducted in patients with esophageal or gastroesophageal junction cancer who were included in the active surveillance arm of the SANO trial. Patients with a CCR following nCRT were selected from the trial database. Only patients who underwent esophagectomy for local regrowth >=6 months after nCRT. Also patients with a strong suspicion of regrowth based on PET-CT scanning and/or nonconclusive biopsies (high grade dysplasia (HGD) or cancer in situ (Tis)) were included. Patients were excluded if the surgical resection specimen was unavailable for review or if no residual tumor (ypT0) was found. As this study used data from the SANO trial (Erasmus MC, MEC-2017-392), no additional ethical approval was required.^10^

### Procedures

Study procedures of the SANO trial have been reported previously.^10^ Briefly, CCR was defined as absence of residual tumor at 3 months after nCRT by PET-CT, endoscopy with multiple bite-on-bite biopsies, and EUS with FNA of suspicious lymph nodes. Patients with CCR underwent surveillance with identical clinical response evaluations (CREs) at 6, 9, 12, 16, 20, 24, 30, 36, 48, and 60 months after nCRT (corresponding to CRE 3 through 12). Surveillance continued until locoregional regrowth and/or distant dissemination was detected. Patients with locoregional regrowth without distant metastases were offered surgical resection.

Resection specimens were reviewed by 2 expert gastro-intestinal (GI)-pathologists, with each assessing approximately half of the cohort. Consensus meetings were held to resolve uncertainties and ensure consistency. Pathology slides (or digital images) were requested from each SANO center. All assessments were performed on hematoxylin-eosin (H&E)–stained sections. Tumor regression grade (TRG) was assessed according to the Mandard scoring system for each esophageal wall layer, including the mucosa, submucosa, proper muscle layer, and surrounding stroma.^11^ In addition, specific regression features were evaluated including, but not limited to, fibrosis, mucin lakes, foreign body giant cells, keratin pearls, and inflammation. Regression patterns were classified as regression toward the lumen (more regression in proper muscle and stroma, residual tumor mainly in mucosa and submucosa), regression toward the invasive front (more regression in mucosa and submucosa, residual tumor mainly in proper muscle and stroma), concentric regression (more regression in mucosa and stroma, residual tumor mainly in submucosa and proper muscle), and random regression (similar regression and residual tumor distribution across all layers). Shapiro et al^12^ presented a schematic representation of regression patterns.

### Outcomes

The primary outcome was the anatomic layer(s) of tumor regrowth within the esophageal wall, with a descriptive evaluation of its distribution with time since completion of nCRT. Secondary outcomes included the regression patterns and a summary of specific regression features observed. It is important to recognize that the absence of tumor in a resection specimen may reflect complete regression after nCRT, but may also result from the fact that not all esophageal wall layers were initially involved by tumor before treatment. To specifically evaluate the biological effect of nCRT on tumor regression, our analysis of regression patterns was therefore restricted to patients with pretreatment T3 tumors in whom all layers were initially at risk for involvement.

### Statistical Analysis

Baseline clinical and tumor characteristics, as well as the site of regrowth within the esophageal wall, were summarized using frequencies and percentages. The association between site of tumor regrowth and regression pattern with time after nCRT and by histology were evaluated. In patients with tumor limited to the submucosa, submucosal depth was classified as SM1, SM2, or SM3, corresponding to invasion of the upper, middle, or lower third of the submucosa. This classification was based on the proportional depth of invasion within the submucosal layer rather than on strict micrometer-based cutoff values. As a practical reference, invasion beyond approximately 500 µm was generally considered SM2 and beyond approximately 1000 µm as SM3.

Interobserver agreement between the 2 pathologists was assessed before consensus meetings on 6 specimens randomly selected by SGP to represent different pathologic T categories, each with 4 points (each layer) of evaluation (24 observations in total). Because this subset was considered representative of the larger cohort, this approach provided a practical estimate of interobserver variability, acknowledging that the remaining patients were assessed by a single observer. We used Cohen’s kappa coefficient, which measures the level of agreement beyond chance. Kappa values were interpreted according to standard benchmarks: values ≤0 indicating no agreement, 0.01–0.20 slight, 0.21–0.40 fair, 0.41–0.60 moderate, 0.61–0.80 substantial, and 0.81–1.00 almost perfect agreement.

Tumor staging was reported according to the 8th edition of the TNM classification. TNM staging from the original pathology reports at each center was compared with the reassessment by the 2 expert GI pathologists and visualized using a Sankey diagram. Statistical analyses were conducted using R version 4.3.2 (R Foundation for Statistical Computing, Vienna, Austria; https://www.R-project.org/).

## RESULTS

In the SANO trial, 191 patients had CCR and entered active surveillance. By December 2023, with a minimum follow-up of 2.5 years, 94 patients had locoregional regrowth 6 to 36 months after nCRT. Of these, 82 underwent transthoracic or transhiatal esophagectomy. For this study, 75 resection specimens were included, with surgery performed at a median of 8 months (IQR 7–12) after nCRT. Seven additional specimens were excluded (Supplementary Fig. S5, Supplemental Digital Content 2, https://links.lww.com/SLA/F991). Regrowth was detected 6 months (CRE 3) after nCRT in 40 patients, 9 months (CRE 4) after nCRT in 15 patients, 12 months (CRE 5) in 10 patients, and in 10 patients later in time (Supplementary Fig. S5, Supplemental Digital Content 2, https://links.lww.com/SLA/F991). The histologic subtype was adenocarcinoma in 60 patients (80%), squamous cell carcinoma in 13 (17%), and adenosquamous carcinoma in 2 patients. Clinical and tumor characteristics are shown in Table 1.

**TABLE 1:** Patient Characteristics

	Study Population (n=75)
Gender	
Female	13 (17)
Male	62 (83)
Mean age in years (SD)	66 (8.5)
Tumor histology	
Adenocarcinoma	60 (80)
Squamous cell carcinoma	13 (17)
Adenosquamous	2 (3)
Grade of differentiation	
G1	14 (19)
G2	38 (51)
G3	23 (31)
Clinical T-category	
cT2	11 (15)
cT3	61 (81)
cTx	3 (4)
Clinical N-category	
cN0	30 (40)
cN1	25 (33)
cN2	16 (21)
cN3	3 (4)
cNx	1 (1)
WHO performance status*	
0	55 (73)
1	15 (20)
Missing	5 (7)
Tumor location†	
Middle esophagus	4 (5)
Distal esophagus	49 (65)
Esophagogastric junction	22 (29)

Values are n (%). Percentages may not add up to 100 because of rounding.

T staging with endoscopic ultrasonography or computed tomography (CT), clinical nodal staging based on endoscopic ultrasonography according to the 8th edition of the Union for International Cancer Control staging manual.

* World Health Organization (WHO) performance-score, assessed on 5-points scale. Higher number indicates greater disability.

† Determined by means of endoscopy.

### Anatomic Distribution of Local Regrowth

Cancer involved the mucosa in 68 of 75 patients (91%). Cancer regrowth was only seen in the submucosa in one patient and in the submucosa and proper muscle layer in another patient (in one patient this was detected by endoscopy with biopsies and another patient by EUS with FNB of the esophageal wall). In one patient, regrowth was confined to the proper muscle layer (endoscopy was suspicious for regrowth, and biopsies were negative but FDG-PET showed increased uptake). In the remaining 4 patients, no invasive tumor was seen in the resection specimen also after reassessment, despite prior biopsies during active surveillance showing HGD/Tis (n=2) or invasive carcinoma (n=2). Reassessment of the specimens are summarized in a Sankey diagram (Supplementary Fig. S4, Supplemental Digital Content 1, https://links.lww.com/SLA/F922). The interobserver agreement for tumor regression in each anatomic layer between the 2 pathologists (MD, LO) was substantial, with a kappa value of 0.79.

In 66 of 75 (88%) patients, regrowth was observed in the submucosa, in the proper muscle layer in 53 of 75 (71%), and in the surrounding stroma in 46 of 75 (61%) (Supplementary Fig. S6, Supplemental Digital Content 3, https://links.lww.com/SLA/F992, Table 2). When regrowth was present in a given layer, it was most often classified as TRG 4 (>50% vital tumor cells) (Supplementary Table S3, Supplemental Digital Content 1, https://links.lww.com/SLA/F922).

**TABLE 2:** Anatomic Distribution of Local Regrowth Per Layer of the Esophageal Wall According to Time After nCRT

Layers involved	Total n=75 (%)	CRE-3 n=40	CRE-4 n=15	CRE-5 n=10	CRE-6 n=2	CRE-8 n=4	CRE-9 n=1	CRE-10 n=3
No (invasive) tumor*	4 (5)	—	2 (13)	—		1		1
M†	4 (5)	3 (8)	—	—			1	
M-SM‡	13 (17)	8 (20)	2 (13)	2 (20)		1		
M-SM-MP	7 (9)	4 (10)	1 (7)	1 (10)		1		
M-SM-MP-S	43 (57)	23 (58)	9 (60)	6 (60)	2	1		2
M-SM-S	1 (1)	1 (3)	—	—				
SM-MP-S	2 (3)	1 (3)	1 (7)	—				
MP	1 (1)	—	—	1 (10)				

Values are n (%). Percentages may not add up to 100 because of rounding.

* Suspected regional lymph node in one patient preoperatively (inconclusive EUS-FNA findings) was ypN0.

† One patients with a positive regional lymph node (N1), which had not been detected on EUS or PET-CT.

‡ Three patients with node positive disease: one N1 (SM1)suspected on PET-CT with uncertain EUS-FNA, and 2 (N1,SM1; N3,SM3) without suspicion on PET-CT or EUS.

CRE indicates clinical response evaluation; M, mucosa; MP, proper muscle layer; S, surrounding stroma; SM, submucosa.

In 4 of 75 patients (5%), cancer regrowth was limited to the mucosa and in 13 of 75 patients (17%), tumor was limited to the mucosa and submucosa. Depth of submucosal invasion was classified as SM1 in 2, SM2 in 2, and SM3 in 9 patients (Supplementary Fig. S6, Supplemental Digital Content 3, https://links.lww.com/SLA/F992, Supplementary Table S2, Supplemental Digital Content 1, https://links.lww.com/SLA/F922). Including the 4 patients without invasive tumor in the resection specimen, 21 of 75 patients (28%) had HGD/Tis or tumor limited to the submucosa. However, regional lymph node metastases were present in 4 of these 21 patients, including one patient with preoperative suspicious nodal disease (Table 2). Thus, 17 of 75 patients (23%) had regrowth limited to the (sub)mucosa without lymph node involvement.

Regrowth according to histology showed that squamous cell carcinoma was confined to the mucosa and/or submucosa in 45% of patients compared with 18% in adenocarcinoma (Supplementary Fig. S1, Supplemental Digital Content 1, https://links.lww.com/SLA/F922, S2, Supplemental Digital Content 1, https://links.lww.com/SLA/F922, Table S1, Supplemental Digital Content 1, https://links.lww.com/SLA/F922). The distribution of cancer regrowth according to anatomic layer was not associated with time after nCRT (Supplementary Fig. S3, Supplemental Digital Content 1, https://links.lww.com/SLA/F922, Supplemental Digital Content 1, https://links.lww.com/SLA/F922, Table 2).

### Regression Pattern

Of the 75 resection specimens, 54 were classified as being initial T3 (pre-pT3) tumors and evaluated for regression patterns. Regression toward the lumen was observed in 15 specimens (28%), concentric regression in 4 (7%), regression toward the invasive front in 4 (7%), and random regression in 29 (54%). In 2 specimens (4%), minimal or no regression was observed. Regression patterns over time were comparable (Supplementary Table S4, Supplemental Digital Content 1, https://links.lww.com/SLA/F922).

### Regression Features

Fibrosis was the most frequently observed regression feature, present across all layers in 87%–95% of specimens. Inflammation occurred mainly in the mucosa (57%) and submucosa (54%) but was also present in the proper muscle layer (28%) and surrounding stroma (24%). In addition, vascular changes such as intimal proliferation, telangiectasia, and organizing thrombi, as well as mucin lakes, were observed, whereas other histologic features were rare (Supplementary Table S5, Supplemental Digital Content 1, https://links.lww.com/SLA/F922).

## DISCUSSION

In this study, we observed that detected cancer regrowth in patients undergoing active surveillance for esophageal carcinoma most frequently involved the mucosa and submucosa (93%). The main regression pattern was random, suggesting that regrowth likely originates from residual cancer cells that were not fully regressed and does not preferentially arise from superficial or deeper tissue layers over time. This supports using endoscopy with bite-on-bite biopsies as a relevant tool in diagnosing regrowth but mucosal involvement does not ensure all regrowth will be detected due to subtle or occult lesions and sampling limitations. In 23% of patients (17 of 75), cancer was confined to the mucosa/submucosa or HGD/Tis without lymph node involvement, suggesting that an endoscopic resection could be an alternative treatment in these patients. However, given the limitations of current diagnostic modalities and the risk of undetected nodal disease, surgery remains the standard of care for regrowth.

Earlier studies were restricted to patients 3 months after nCRT and demonstrated that residual disease was most often located in the mucosa and submucosa.^3,12^ Our findings suggest that this pattern is also seen in patients with regrowth up to 36 months after nCRT, although patient numbers were limited at later time points and not all patients underwent surgery. Chao et al^13^ reported residual tumor beneath tumor-free mucosa in 22% of patients (16 of 73 patients) with squamous cell carcinoma and CCR at 4–6 weeks post-nCRT. We could not confirm this observation in our smaller subgroup (n=13) of patients with squamous cell carcinoma and this certainly needs further study.

Structural alterations of the esophageal wall 3 months after nCRT can complicate endoscopic detection and make it difficult to distinguish HGD/Tis from intramucosal carcinoma (T1a) due to post-treatment mucosal atrophy and fibrosis.^3^ In addition, for adenocarcinoma, glandular structures may mimic invasive growth. In our experience, this diagnostic challenge persists even beyond 3 months after nCRT.^14^ Moreover, in resection specimens the pathologic assessment of T1a versus T1b tumors can be inaccurate, since the muscularis mucosae is often obscured due to the treatment induced fibrosis and atrophy.^3^

The accuracy of EUS for assessing the depth of local regrowth is limited due to treatment-related fibrosis and distortion of the esophageal wall after nCRT.^15^ A diagnostic endoscopic resection will provide the most accurate determination of tumor depth.^16^ ESD allows complete resection of the submucosa and has already been successfully performed as salvage therapy after definitive CRT, achieving curative resection rates of 58–100% and low complication rates of 0–3%.^6–8,17^ Although the majority of patients in these studies had squamous cell carcinoma, these results indicate that the procedure is technically feasible, and may also be applicable in adenocarcinoma.^7,8^

When considering endoscopic resections for local regrowth, the risk of undetected nodal disease must be recognized as current response evaluation cannot exclude occult nodal disease. The risk of lymphatic spread increases with deeper submucosal invasion (particularly SM3) and poor tumor differentiation.^18,19^ Most patients with tumor regrowth limited to the submucosa in our cohort had SM3 involvement (9 of 13). Whether the prognostic relevance of submucosal depth after nCRT is comparable to the situation before nCRT is unclear. Of the 28 patients with tumor limited to the mucosa or submucosa, 4 patients had nodal involvement and in 3 patients nodal involvement was not diagnosed before surgery. This highlights the risk of missing nodal disease during active surveillance and, at present, do not support the use of endoscopic resection. If patients with HGD/Tis or T1 cancer decline surgery despite recommendation, serial follow-up after endoscopic resection would be necessary to detect regional lymph node metastases, as in surveillance for high-risk T1 cancer. However, the risk of missed nodal disease should be discussed with the patient.^16^

Shapiro et al^12^ reported primarily luminal and concentric regression within 3 months after nCRT. In contrast, the predominant regression pattern in our study was random and this remained consistent over time. This suggests that after nCRT, (small clusters of) vital tumor cells may reside in the esophageal wall. Consequently, local regrowth during active surveillance should by principle be considered residual disease. The random regression pattern observed in our study could be due to continued regression in superficial layers, altering the pattern. However, this could not be observed during evaluation of the pathologic specimen. As the mucosa is also involved in random regression, endoscopy might capture residual tumor just as effectively as with regression toward the lumen.

Although prior studies suggested tumor regression might continue for up to 3 months after nCRT, the NeoRes trial found no increase in complete pathologic response with longer intervals.^20^ If regression is maximum within 3 months, the predominance of random regression could explain why residual disease was missed at CRE-1 and CRE-2. Most cases of local regrowth were detected at CRE-3 (6 months post-nCRT), which may reflect sampling limitations rather than differences in tumor biology. We therefore do not consider these early regrowths to represent aggressive disease, but rather missed residual tumor that was difficult to detect shortly after nCRT. Strict adherence to the surveillance schedule may be difficult to achieve in routine clinical practice, and any deviations could further limit the safety and effectiveness of active surveillance.

This study is the first to assess patterns of local regrowth in patients with initial CCR 3 months post-nCRT undergoing active surveillance. Repeated assessments are a key strength, and the predominantly random regrowth pattern suggests residual disease is unlikely to remain undetected for long, though sampling errors remain a limitation. Several limitations should be noted. A limitation of this study is that outcomes for patients who did not proceed to surgery due to refusal, unresectable disease, being unfit for surgery, or metastases are unknown, reflecting inherent selection bias. For most, a different regrowth pattern is unlikely, and in those with metastases but no detected locoregional regrowth, the majority (22 of 32) occurred within 6 months after nCRT, suggesting systemic involvement was already present. Furthermore, the majority of residual disease was detected at CRE-3 (6 months after nCRT) and patient numbers at later time points were limited. Outcome for time points beyond 6 months should be interpreted with caution. This also applies to the subgroup of patients with squamous cell carcinoma, which included only 13 patients. Moreover, Mandard TRG was used to assess regrowth patterns, but its long-term relevance after nCRT is uncertain, it still allows hypotheses on residual disease distribution. Lastly, not all specimens were reviewed by both pathologists, although consensus review and the pathologists’ extensive experience support the reliability of the findings.

In conclusion, in most patients with detected local regrowth after nCRT for esophageal carcinoma, tumor involvement included the mucosa and submucosa and layer distributions did not change over time, suggesting that endoscopy with multiple biopsies remains an important detection procedure during response evaluations. Given the risk of undetected nodal disease and the current limitations of staging after nCRT, surgery remains the recommended treatment for cancer regrowth.

## Supplementary Material

## DISCUSSANTS

### Philippe Nafteux (Leuven, Belgium)

Thank you very much. I would like to thank the ESA for the opportunity to be first discussant of this very interesting paper. The current paper is a follow-up study of the SANO trial looking for assurance at the pathologic level for recurrence patterns and location. The clinical motivation is obviously clear. Organ-sparing treatment could be a viable future strategy as long as timely detection remains possible. If regrowth is predominantly local, at the mucosal or submucosal layers, then it sounds possible, and endoscopic surveillance should be the key for the follow-up. The methods were clear, the evaluation process thorough, and the conclusion straightforward. The majority of patients have mucosal or submucosal recurrence, so basically this seems to be a very good foundation for a surveillance program. Nevertheless, I have some remarks and questions. Let’s talk about the selection bias. To what extent do the findings in your evaluation really represent real-life, daily practice, taking into account the selection bias of the SANO trial itself (low T, low number of positive lymph nodes as compared to, for example, the ESOPEC trial, where patients have higher T and N factors) and the exclusion of patients who actually will not be operated on? Furthermore, you started with 75 resection specimens; however, for the regression analysis, you only looked at 54 of them, as you were only looking at the cT3 patients. Basically, you end up with a very small number of patients.

Let’s talk about the protocol: when you perform an endoscopy after chemoradiation therapy, you are not always aware of what you are looking at, meaning that a lot of these residual mucosal lesions are in the middle of scar tissue. Biopsies are not very accurate, and sampling errors are very common. EUS is also not very reliable in detecting recurrence. It is worrisome that about 70% of your patients, if I recall what was stated in the manuscript correctly, will be at least T2 or T3, and 40-60% will have positive lymph nodes. This raises an important question. Is this really due to a sampling error, or the more aggressive biology of the remaining disease, resulting in a deleterious prognosis for the patients? If it’s the latter, could it be a missed opportunity? In the SANO trial, you look at 2-year survival curves. At 2 years, survival is in favor of the surgical group. Most likely, you will need a follow-up study based on 5-year survival, in order to provide the answer to this.

Finally, this surveillance protocol could be quite important, and I understand that having the possibility to avoid an esophagectomy is quite tempting, especially for patients. However, in order to be able to achieve this goal, you need a strict follow-up protocol, which is labor intensive and time-consuming. Currently, all endoscopy suites are fully booked and have a really burdensome workload. The same can be said about the pathology department. So, from a pragmatic point of view, how do you envisage such a rigorous protocol to be implemented in daily clinical routine?

### Response From Sanjiv Gangaram Panday (Rotterdam, The Netherlands)

Thank you for your questions and for your thorough review. We agree that the main limitation of this study is the potential selection bias in patients who developed distant metastases, as we cannot determine whether an unrecognized local regrowth contributed to the metastatic disease. The same applies to the 2 patients with unresectable local regrowth. Following your comment, we re-examined these cases and found that, of the 32 patients who developed distant metastases during follow-up, 21 already had metastases detected within 6 months after chemoradiotherapy, suggesting that metastatic disease was already likely present. This leaves 10 patients, together with the 2 patients with unresectable local regrowth, for whom we cannot exclude the possibility that their regression pattern – for example, regression toward the invasive front – may have influenced the outcome. This represents a potential source of selection bias that cannot be corrected for in our analysis.

Regarding your following question, we believe this is primarily due to a sampling error. As shown in the pre-SANO trial, biopsies may miss patients with tumor regression grade (TRG) 2, whereas the main objective was to avoid missing patients with TRG3 or TRG4 residual disease. Therefore, patients with TRG2 may not have been detected at the second clinical response evaluation (CRE2), and identified instead at CRE3. This is consistent with the random regression pattern after chemoradiotherapy, where biopsies can easily miss residual tumor. Regarding the higher rate of lymph node involvement, we believe this reflects the selection bias, as our analysis only included patients with residual tumors, who developed local regrowth. A fair comparison with the standard surgery cohort would also include patients with a sustained clinical complete response. In that case, we expect the proportion of node-negative (N0) disease to be comparable between the groups.

Finally, the standardization of clinical response evaluation is essential in a trial setting, and the ongoing SANO-2 study is prospectively evaluating the quality of these assessments across participating hospitals and whether variation in response evaluation affects overall survival. We also believe the response evaluation strategy can be further optimized. In particular, we found that endoscopic ultrasound (EUS) has limited additional value for detecting lymph node involvement when a PET/CT is performed first and EUS is reserved for selected indications. Reducing the routine use of EUS could help alleviate the burden on endoscopy services. Although active surveillance will continue to require more endoscopic procedures, we believe this is justified by the opportunity to spare selected patients an esophagectomy.

### Richard van Hillegersberg (Utrecht, The Netherlands)

I think one of the main problems we’re facing is the lack of a good response evaluation following neoadjuvant therapy.

You showed that about 50% of patients who were considered to have achieved a clinical complete response subsequently developed tumor recurrence; yet, you consider that this is probably not harmful.

However, at the same time, we know that the NeoRes trial showed that delayed surgery may result in a higher rate of metastasis. Also, in your primary SANO trial paper, there’s a higher rate of distant metastasis in the wait-and-see group.

Therefore, I do not think that you can conclude that a wait-and-see policy is not harmful in patients where you have a 50% recurrence rate.

### Response From Sanjiv Gangaram Panday (Rotterdam, The Netherlands)

Thank you for your comments. The NEORES study demonstrated that delaying surgery from 6 to 12 weeks does not increase the pathological complete response rate. Although an unadjusted subgroup analysis suggested worse outcomes in the small subgroup of patients with tumor regression grade (TRG) 4 (23 versus 21 patients), these findings should be interpreted with caution, particularly as other studies have not shown an adverse effect of delayed surgery. Therefore, we believe delayed surgery remains safe, although the higher rate of distant metastases observed warrants a continued follow-up. The 5-year results will be essential to determine whether this translates into a difference in overall survival. Regarding the suggestion to correlate histopathology with the initial endoscopic findings, we reviewed a subset of pathology slides together with the pathologist. However, a meaningful analysis would require an evaluation of the entire cohort rather than only patients with residual disease, as this would enable a direct comparison with patients without residual tumors. Using the currently available diagnostic tools, we did not identify any additional features that could explain the failures or warrant further investigation.

### Pawel Mroczkowski (Bochum, Germany)

Is this approach currently limited to the setting of a clinical trial, or do you usually offer this wait-and-see approach to a normal patient? In other words, do you routinely wait 3 months after neoadjuvant therapy to decide whether to operate or not?

### Response From Sanjiv Gangaram Panday (Rotterdam, The Netherlands)

Yes. Following the SANO trial, we started the SANO-2 cohort, which is a prospective cohort study. Patients undergo clinical response evaluations at 6 and 12 weeks after neoadjuvant chemoradiotherapy. If they have a clinical complete response, they are offered active surveillance.

During active surveillance, patients are followed every 3 months in the first year, every 4 months in the second year, every 6 months in the third year, and annually in years 4 and 5. At each follow-up visit, the same clinical response evaluation is performed, consisting of a PET/CT, endoscopy, and EUS.

Patients remain in this prospective cohort because we are still awaiting the long-term, 5-year outcomes of active surveillance.
