# Supplementary material for: Local Regrowth After Neoadjuvant Chemoradiotherapy and Active Surveillance for Esophageal Cancer: An Analysis of Surgical Specimens From the SANO Trial
Source: Ann Surg. 2026 Aug 21;284(5):964–70. doi: 10.1097/SLA.0000000000007179 (PMC13652127; doi:10.1097/SLA.0000000000007179)
Supplement: Supplementary file 1 [file sla-284-0964-s001.docx]

| **Table of content** | **Page** |
| --- | --- |
| Figure S1 Anatomical distribution of local regrowth per layer of the esophageal wall for adenocarcinoma | 2 |
| Figure S2 Anatomical distribution of local regrowth per layer of the esophageal wall for squamous cell carcinoma | 3 |
| Figure S3 Anatomical distribution of local regrowth per layer of the esophageal wall over time | 4 |
| Table S1 Anatomical distribution of local regrowth per layer of the esophageal wall according to histology | 5 |
| Table S2 Pathology of the resection specimens with reassessment | 6 |
| Table S3 Tumor regression grade per layer of the esophageal wall | 7 |
| Table S4 Regression pattern of the esophageal wall according to time after nCRT | 8 |
| Table S5 Regression features per layer of the esophageal wall with tumor or tumor regression | 9 |
| Figure S4 Reassessment of T-category and tumor regression grade (TRG) | 10 |
| Table S6 Members of the SANO study group | 11 |

Figure S1 Anatomical distribution of local regrowth per layer of the esophageal wall for adenocarcinoma (n=60). Each column in the bottom chart represents one patient. The first two columns on the left represent two patients without invasive tumor in the resection specimen.

Figure S2 Anatomical distribution of local regrowth per layer of the esophageal wall for squamous cell carcinoma (n=13). Each column in the bottom chart represents one patient. The first two columns on the left represent two patients without invasive tumor in the resection specimen.

**CRE5**

**CRE3**

**CRE4**

Figure S3 Anatomical distribution of local regrowth per layer of the esophageal wall for CRE3 (n=40), CRE4 (n=15) and CRE5 (n=10).

**Table** S1 Anatomical distribution of local regrowth per layer of the esophageal wall according to histology

| Layers involved | Total n=75 (%) | EAC n=60 | ESCC n=13 |
| --- | --- | --- | --- |
| **No (invasive) tumor^1^** | 4 (5) | 2 (3) | 2 (15) |
| **M^2^** | 4 (5) | 3 (5) | 1 (7) |
| **M-SM^3^** | 13 (17) | 8 (13) | 5 (38) |
| **M-SM-MP** | 7 (9) | 6 (10) | 1 (8) |
| **M-SM-MP-S** | 43 (57) | 38 (63) | 3 (23) |
| **M-SM-S** | 1 (1) | 1 (2) | - |
| **SM-MP-S** | 2 (3) | 1 (2) | 1 (8) |
| **MP** | 1 (1) | 1 (2) | - |
| Values are n (%). Percentages may not add up to 100 because of rounding. EAC, esophageal adenocarcinoma; ESCC, esophageal squamous cell carcinoma; CRE, clinical response evaluation; M, mucosa; SM, submucosa; MP, proper muscle layer; S, surrounding stroma.  ^1^suspected regional lymph node in one patient preoperatively (inconclusive EUS-FNA findings) was ypN0.  ^2^one patients with a positive regional lymph node (N1), which had not been detected on EUS or PET-CT.  ^3^three patients with node positive disease: one N1 (SM1)suspected on PET-CT with uncertain EUS-FNA, and two (N1,SM1; N3,SM3) without suspicion on PET-CT or EUS. | | | |

Table S2 Pathology of the resection specimens with reassessment

|  | Study population (n=75) | Reassessment* |
| --- | --- | --- |
| **Histological grading** |  |  |
| G1 | 2 (3) |  |
| G2 | 53 (71) |  |
| G3 | 7 (9) |  |
| G4 | 10 (13) |  |
| Gx | 1 (1) |  |
| No primary tumour present | 2 (3) |  |
| **Tumour regression grade** |  |  |
| TRG1 | 2 (3) | 4 (5) |
| TRG2 | 7 (9) | 7 (9) |
| TRG3 | 23 (31) | 18 (24) |
| TRG4 | 42 (56) | 46 (61) |
| Missing | 1 (1) | 0 (0) |
| **ypT** |  |  |
| Tis | 2 (2) | 3 (4) |
| 0 | 0 (0) | 1 (1) |
| 1 | 17 (23) |  |
| 1a | - | 4 (5) |
| 1b | - | 13 (17)^1^ |
| 2 | 10 (13) | 8 (10) |
| 3 | 44 (59) | - |
| 4a | 2 (3) | - |
| 3/4a |  | 46 (61) |
| **ypN** |  |  |
| 0 | 44 (59) |  |
| 1 | 19 (25) |  |
| 2 | 9 (12) |  |
| 3 | 3 (4) |  |
| **ypT0N+** | 0 (0) | 0 (0) |
| **ypT1aN1** | - | 1 (1) |
| **ypT1bN+** | - | 3 (4)^2^ |
|  |  |  |
| **Median time in months after nCRT (IQR)** | 8.4 (6.9-11.7) |  |
| Values are n (%). Percentages may not add up to 100 because of rounding. cTNM staging was indicated according to the 8^th^ edition of the Union for International Cancer Control staging manual. TRG (tumor regression grade) was indicated according to the Mandard’s classification. ^1^2xSM1, 2xSM2, 9xSM3; ^2^2x N1, 1xN3. *reassessment by study center’s pathologist | | |

Table S3 Tumor regression grade per layer of the esophageal wall

| Total n=75 (%) | Mucosa | Submucosa | Proper  muscle layer | Stroma |
| --- | --- | --- | --- | --- |
| **TRG1** | 7 (9) | 8 (11) | 14 (19) | 8 (11) |
| **TRG2** | 11 (15) | 5 (7) | 9 (12) | 8 (11) |
| **TRG3** | 13 (17) | 21 (28) | 6 (8) | 10 (13) |
| **TRG4** | 36 (48) | 36 (48) | 33 (44) | 26 (35) |
| **TRG5** | 8 (11) | 4 (5) | 5 (7) | 2 (3) |
| **NA** | - | 1 (1) | 8 (11) | 21 (28) |
| NA: no tumor or regression present; TRG, tumor regression grade  Values are n (%). | | | | |

Table S4 Regression pattern of the esophageal wall according to time after nCRT

| Regression Pattern | Total n=54 (%) | CRE-3 n=29 | CRE-4 n=11 | CRE-5 n=8 | CRE-6 n=2 | CRE-8  n=2 | CRE-10 n=2 |
| --- | --- | --- | --- | --- | --- | --- | --- |
| **Regression toward the lumen** | 15 (28) | 9 (31) | 3 (27) | 2 (25) |  |  | 1 |
| **Concentric regression** | 4 (7) | 2 (7) |  | 2 (25) |  |  |  |
| **Regression toward the invasive front** | 4 (7) | 2 (7) | 2 (18) |  |  |  |  |
| **Random regression** | 29 (54) | 15 (52) | 5 (45) | 4 (50) | 2 | 2 | 1 |
| **No regression** | 2 (4) | 1 (3) | 1 (9) |  |  |  |  |
| Values are n (%). Percentages may not add up to 100 because of rounding. CRE, clinical response evaluation. | | | | | | | |

|  | Mucosa  n=75 | Submucosa  n=74 | Proper muscle  layer n=67 | Stroma  n=54 |
| --- | --- | --- | --- | --- |
| Fibrosis | 65 (87) | 70 (95) | 62 (93) | 51 (94) |
| Mucin pools | 6 (8) | 10 (14) | 5 (7) | 8 (15) |
| Inflammation | 43 (57) | 40 (54) | 19 (28) | 13 (24) |
| Intimal proliferation of vessels/  telangiectasia/organizing thrombi | 1 (1) | 13 (18) | 1 (1) | 16 (30) |
| Atrophy of submucosal glands | NA | 8 (11) | NA | NA |
| Values are n (%). NA, not applicable. | | | | |

Table S5 Regression features per layer of the esophageal wall with tumor or tumor regression

Figure S4 Reassessment of T-category and tumor regression grade (TRG); In four patients with no tumor detected in the resection specimen after reassessment, initial evaluation had indicated HGD in one specimen, which was revised to no residual tumor (fibrotic area only), and T1 carcinoma in the remaining three, which was revised to HGD upon reassessment.

Table S6 Members of the SANO study group, besides the authors who are indicated on the title page

| Name | Department | Center |
| --- | --- | --- |
| Arjun D. Koch | Gastroenterology | Erasmus MC – University medical center |
| Suzan Nikkessen | Gastroenterology | Erasmus MC – University medical center |
| Ate van der Gaast | Medical oncology | Erasmus MC – University medical center |
| Roelf Valkema | Nuclear medicine | Erasmus MC – University medical center |
| Hester F. Lingsma | Public health | Erasmus MC – University medical center |
| David van Klaveren | Public health | Erasmus MC – University medical center |
| Roy S. Dwarkasing | Radiology | Erasmus MC – University medical center |
| Berend van der Wilk | Surgery | Erasmus MC – University medical center |
| Ben Eyck | Surgery | Erasmus MC – University medical center |
| Bo Jan Noordman | Surgery | Erasmus MC – University medical center |
| Maartje Valkema | Surgery | Erasmus MC – University medical center |
|  |  |  |
| Geert J. Bulte | Gastroenterology | Radboud University Medical Center |
| Peter D. Siersema | Gastroenterology | Radboud University Medical Center |
| Tanya M. Bisseling | Gastroenterology | Radboud University Medical Center |
| Harm Westdorp | Medical oncology | Radboud University Medical Center |
| Erik H. Aarntzen | Nuclear medicine | Radboud University Medical Center |
| Maartje C. van Rijk | Radiology | Radboud University Medical Center |
| Pètra M. Braam | Radiotherapy | Radboud University Medical Center |
| Heidi Rütten | Radiotherapy | Radboud University Medical Center |
| Marcel Verheij | Radiotherapy | Radboud University Medical Center |
| Camiel Rosman | Surgery | Radboud University Medical Center |
| Bastiaan Klarenbeek | Surgery | Radboud University Medical Center |
|  |  |  |
|  |  |  |
| Serge J. Zweers | Gastroenterology | Maasstad Hospital |
| Lisanne Holster | Gastroenterology | Maasstad Hospital |
| Lieke Hol | Gastroenterology | Maasstad Hospital |
| Ewout F.W. Courrech Staal | Radiology | Maasstad Hospital |
| Trudy Rapmund | Surgery | Maasstad Hospital |
| Erwin van der Harst | Surgery | Maasstad Hospital |
| Peter-Paul Coene | Surgery | Maasstad Hospital |
|  |  |  |
| Peter Oosterwijk | Gastroenterology | Ziekenhuisgroep Twente Hospital |
| Polat Dura | Gastroenterology | Ziekenhuisgroep Twente Hospital |
| Andries van der Linden | Gastroenterology | Ziekenhuisgroep Twente Hospital |
| Ronald Hoekstra | Medical oncology | Ziekenhuisgroep Twente Hospital |
| Ali Agool | Nuclear medicine | Ziekenhuisgroep Twente Hospital |
| Ellen M. Hendriksen | Radiotherapy | Ziekenhuisgroep Twente Hospital |
| Henk Jan Mantel | Surgery | Ziekenhuisgroep Twente Hospital |
| Marc van Det | Surgery | Ziekenhuisgroep Twente Hospital |
| Ewout Kouwenhoven | Surgery | Ziekenhuisgroep Twente Hospital |
|  |  |  |
| Sana A. Mulder | Gastroenterology | Reinier de Graaf Gasthuis |
| Rutger Quispel | Gastroenterology | Reinier de Graaf Gasthuis |
| Arjan J. Verschoor | Medical oncology | Reinier de Graaf Gasthuis |
| Marc R.J. ten Broek | Nuclear medicine | Reinier de Graaf Gasthuis |
| René J. Dallinga | Radiology | Reinier de Graaf Gasthuis |
| Karen J. Neelis | Radiotherapy | Reinier de Graaf Gasthuis |
| Erlinde de Graaf | Surgery | Reinier de Graaf Gasthuis |
| Stijn van Esser | Surgery | Reinier de Graaf Gasthuis |
| Jan Willem Dekker | Surgery | Reinier de Graaf Gasthuis |
|  |  |  |
| Thomas R. de Wijkerslooth | Gastroenterology | The Netherlands Cancer Institute |
| Jolanda M. van Dieren | Gastroenterology | The Netherlands Cancer Institute |
| Marieke A. Vollebergh | Medical oncology | The Netherlands Cancer Institute |
| Emilia C. Owers | Nuclear medicine | The Netherlands Cancer Institute |
| Annemarieke Bartels-Rutten | Radiology | The Netherlands Cancer Institute |
| Francine E.M. Voncken | Radiotherapy | The Netherlands Cancer Institute |
| Yvonne Hilhorst | Surgery and gastroenterology | The Netherlands Cancer Institute |
| Marjolein Warmerdam | Surgery and gastroenterology | The Netherlands Cancer Institute |
| Johanna van Sandick | Surgery | The Netherlands Cancer Institute |
|  |  |  |
| Sietske Corporaal | Gastroenterology | Medical Center Leeuwarden |
| Edward Fiets | Medical oncology | Medical Center Leeuwarden |
| Marco B. Polée | Medical oncology | Medical Center Leeuwarden |
| Anne Marij G. van Burg | Nuclear medicine | Medical Center Leeuwarden |
| Rinze Wolf | Radiology | Medical Center Leeuwarden |
| Vera Oppedijk | Radiotherapy | Medical Center Leeuwarden |
| Marloes Emous | Surgery | Medical Center Leeuwarden |
| Daniel A. Hess | Surgery | Medical Center Leeuwarden |
| Jean Pierre Pierie | Surgery | Medical Center Leeuwarden |
|  |  |  |
| Willemien Erkelens | Gastroenterology | Gelre Hospital |
| Eva Kouw | Gastroenterology | Gelre Hospital |
| S. Cathrien S. Tromp – van Driel | Medical oncology | Gelre Hospital |
| Marc D. Zuijdwijk | Nuclear medicine | Gelre Hospital |
| Karin Muller | Radiotherapy | Gelre Hospital, Radiotherapiegroep Deventer |
| Peter van Duijvendijk | Surgery | Gelre Hospital |
| Eelco B. Wassenaar | Surgery | Gelre Hospital |
| Edwin van der Zaag | Surgery | Gelre Hospital |
|  |  |  |
| Erik J. Schoon | Gastroenterology | Catharina Hospital |
| Geert-Jan Creemers | Medical oncology | Catharina Hospital |
| Mark J. Roef | Nuclear medicine | Catharina Hospital |
| Joost Nederend | Radiology | Catharina Hospital |
| Maurice J.C. van der Sangen | Radiotherapy | Catharina Hospital |
| Tom C.G. Budiharto | Radiotherapy | Catharina Hospital |
| Fanny F.B.M. Heesakkers | Surgery | Catharina Hospital |
| Misha Luyer | Surgery | Catharina Hospital |
| Grard A. P. Nieuwenhuijzen | Surgery | Catharina Hospital |
|  |  |  |
| Liekele E. Oostenbrug | Gastroenterology | Zuyderland Medical Center |
| Fabienne A.R.M. Warmerdam | Medical oncology | Zuyderland Medical Center |
| Wendy Schreurs | Nuclear medicine | Zuyderland Medical Center |
| Roy F.A. Vliegen | Radiology | Zuyderland Medical Center |
| Jeroen Buijsen | Radiotherapy | Zuyderland Medical Center |
| Ilse Stohr | Surgery | Zuyderland Medical Center |
| Eric H.J. Belgers | Surgery | Zuyderland Medical Center |
| Meindert Sosef | Surgery | Zuyderland Medical Center |
|  |  |  |
| Jurjen J. Boonstra | Gastroenterology | Leiden University Medical Center |
| Marije Slingerland | Medical oncology | Leiden University Medical Center |
| Richard Raghoo | Nuclear medicine | Leiden University Medical Center |
| A. Stijn L.P. Crobach | Pathology | Leiden University Medical Center |
| Aart J. van der Molen | Radiology | Leiden University Medical Center |
| Susan J.C.L.M. Quix | Surgery | Leiden University Medical Center |
| Wobbe O. de Steur | Surgery | Leiden University Medical Center |
| Henk Hartgrink | Surgery | Leiden University Medical Center |
|  |  |  |
| Wouter L. Hazen | Gastroenterology | Elisabeth Tweesteden Hospital |
| Laurens V. Beerepoot | Medical oncology | Elisabeth Tweesteden Hospital |
| Tom Rozema | Radiotherapy | Elisabeth Tweesteden Hospital |
| Ilse A.C. Vermeltfoort | Nuclear medicine | Elisabeth Tweesteden Hospital |
| Walther Jansen | Surgery | Elisabeth Tweesteden Hospital |
| Joos Heisterkamp | Surgery | Elisabeth Tweesteden Hospital |
